# Identification of a novel role for matrix metalloproteinase-3 in the modulation of B cell responses in multiple sclerosis

**DOI:** 10.3389/fimmu.2022.1025377

**Published:** 2022-10-26

**Authors:** Rittika Chunder, Verena Schropp, Samir Jabari, Manuel Marzin, Sandra Amor, Stefanie Kuerten

**Affiliations:** ^1^ Institute of Neuroanatomy, Medical Faculty, University of Bonn, Bonn, Germany; ^2^ Institute of Anatomy and Cell Biology, Friedrich-Alexander-Universität Erlangen-Nürnberg (FAU), Erlangen, Germany; ^3^ Institute of Neuropathology, University Hospitals Erlangen, Erlangen, Germany; ^4^ Department of Pathology, Amsterdam University Medical Center, Amsterdam, Netherlands

**Keywords:** B cells, central nervous system, immune modulation, matrix metalloproteinase-3, multiple sclerosis

## Abstract

There has been a growing interest in the presence and role of B cell aggregates within the central nervous system of multiple sclerosis patients. However, very little is known about the expression profile of molecules associated with these aggregates and how they might be influencing aggregate development or persistence in the brain. The current study focuses on the effect of matrix metalloproteinase-3, which is associated with B cell aggregates in autopsied multiple sclerosis brain tissue, on B cells. Autopsied brain sections from multiple sclerosis cases and controls were screened for the presence of CD20^+^ B cell aggregates and expression of matrix metalloproteinase-3. Using flow cytometry, enzyme-linked immunosorbent assay and gene array as methods, *in vitro* studies were conducted using peripheral blood of healthy volunteers to demonstrate the effect of matrix metalloproteinase-3 on B cells. Autopsied brain sections from multiple sclerosis patients containing aggregates of B cells expressed a significantly higher amount of matrix metalloproteinase-3 compared to controls. *In vitro* experiments demonstrated that matrix metalloproteinase-3 dampened the overall activation status of B cells by downregulating CD69, CD80 and CD86. Furthermore, matrix metalloproteinase-3-treated B cells produced significantly lower amounts of interleukin-6. Gene array data confirmed that matrix metalloproteinase-3 altered the proliferation and survival profiles of B cells. Taken together, out data indicate a role for B cell modulatory properties of matrix metalloproteinase-3.

## 1 Introduction

Multiple sclerosis (MS) is a chronic neuroinflammatory demyelinating disease of the central nervous system (CNS) (1). The multifaceted roles of B cells in the pathogenesis of MS have gained increasing attention over the last years (2, 3), with one of the highlights being the clinical success story of peripheral blood B cell depletion with monoclonal anti-CD20 antibodies in relapsing-remitting (4, 5) and primary progressive MS patients (6, 7). Additional evidence for a key role of B cells in MS comes from neuropathological studies on autopsied brain sections (8, 9). On the one hand, studies that were performed more than a decade ago identified the presence of lymphoid-like B cell follicles in the inflamed meninges of up to 40% of MS patients with the secondary progressive disease form and observed a correlation with more severe clinical disease and cortical pathology (10–13). On the other hand, CD20^+^ B cell infiltrates were also found in primary progressive MS patients (14) and more recently, a prominent presence of B cells in lesions of patients with acute MS was demonstrated by post-mortem studies (15). While it has been suggested that these lymphoid-like B cell follicles may be drivers of disease progression (11–14, 16–19) the molecular signature associated with the presence of B cells within the CNS lesions and the triggers that convert B cells into pathogenic entities have not yet been clearly elucidated (20, 21).

To model the contribution of B cells in the human disease we immunized C57BL/6 mice with MP4 – a fusion protein consisting of the human isoform of myelin basic protein (MBP) and the three hydrophilic domains of proteolipid protein (PLP) (22). B cell aggregates resembling ectopic lymphoid-structures were predominantly observed in the cerebellum of MP4-immunized mice in the chronic stage of the disease (23, 24). To identify key molecules associated with or expressed within these follicles, gene expression profiles of B cell aggregates isolated from MP4-immunized mice were analyzed and led to the identification of matrix metalloproteinase (MMP)-3 (25), a member of the matrix metalloproteinase family. MMPs are pleiotropic proteases traditionally associated with the degradation and turnover of components of the extracellular matrix (ECM) (26). More recent evidence suggests that only 27% of MMP substrates are ECM or ECM-related proteins, while the majority of the known MMP substrates are non-ECM proteins including chemokines, cytokines, cell-surface receptors and proteins involved in immune signaling (27). An extension of these new findings is also a shift in the paradigm for MMP functions from being only destructive enzymes that are detrimental in inflammatory diseases to being regarded as cell signaling regulators and modulators of inflammatory and immune processes (28, 29).

In the context of neuroinflammation and MS, MMPs have been implicated both as positive and negative regulators of tissue injury (30–34). For instance, MMP-3 has been shown to degrade protein components that inhibit differentiation and maturation of oligodendrocytes (35) and to terminate inflammation within the CNS (36). Conversely, other reports have discussed the unfavorable role of MMP-3 as a driving factor for neuroinflammation and neurodegeneration (26, 37), including its association with increased blood-brain barrier (BBB) permeability (38–40).

To our knowledge, this study is the first to have addressed the relationship between MMP-3 and B cells. Based upon the preliminary data obtained from the MP4-induced experimental autoimmune encephalomyelitis (EAE) mouse model where gene expression of MMP-3 was spatially associated with the presence of B cell aggregates (25), here we analyzed the expression pattern of MMP-3 in MS brain tissue. Our data demonstrate a high level of MMP-3 expression especially in association with B cell aggregates. When incubating B cells of peripheral human blood with human recombinant MMP-3 (rMMP-3) we observed downregulated B cell activation and cytokine production, in particular of the pro-inflammatory cytokine interleukin-6 (IL-6). These data imply a favorable role of MMP-3 in polarizing B cells to a less pathogenic phenotype.

## 2 Materials and methods

### 2.1 Human brain tissue

Paraffin-embedded brain tissue sections of MS patients and control subjects were obtained from the Multiple Sclerosis and Parkinson’s Tissue Bank, Center for Brain Sciences, Imperial College London (approved by the Ethics Committee of the University of Würzburg, File 258/14) and the Department of Pathology, Amsterdam University Medical Center. Human brain tissue was obtained at autopsy using the rapid autopsy regimen of the Netherlands Brain Bank (co-ordinator Prof. I. Huitiga), Amsterdam, with approval of the Medical Ethical Committee of the Amsterdam UMC. All participants or next of kin had given informed consent for autopsy and use of tissues for research purposes. MS brain samples that were obtained from the Netherlands Brain Bank were selected from regions of interest (ROIs) after *ex vivo* MRI to detect lesions. Tissue blocks containing the ROI were cut in half and half fixed in 10% formalin and embedded in paraffin and the other half snap-frozen and stored in liquid nitrogen. General information and clinical details on the patient samples are listed in **Table 1**. 16 cases of MS and 15 controls were included in the study.

**Table 1:** Patient details.

MS patients							
Number	Age	Sex	Multiple sclerosis type	Disease duration in years	Cause of death	Number of blocks	Patient type
MS325	51	m	Primary progressive	2	Bronchopneumonia	2	NI
MS342	35	f	Secondary progressive	5	Multiple sclerosis	2	B cell
MS402	46	m	Secondary progressive	20	Multiple sclerosis, bronchopneumonia	1	B cell
MS407	44	f	Secondary progressive	19	Sepsis, pneumonia	1	B cell
MS408	39	m	Secondary progressive	10	Sepsis, pneumonia	1	NI
MS438	53	f	Unknown	18	Multiple sclerosis	2	NI
MS444	49	m	Secondary progressive	20	Kidney failure	2	T cell
MS473	39	f	Primary progressive	13	Multiple sclerosis, bronchopneumonia	2	T cell
MS485	57	f	Primary progressive	29	Multiple sclerosis, bronchopneumonia	2	T cell
MS510	38	f	Secondary progressive	22	Multiple sclerosis, pneumonia	1	T cell
MS523	63	f	Secondary progressive	32	Bronchopneumonia	1	T cell
MS528	45	f	Secondary progressive	25	Pneumonia	1	T cell
11-077	66	f	Primary progressive	32	Euthanasia	5	B cell
14-006	56	f	Unknown	31	Suicide by pentobarbital intoxication	2	B cell
15-047	50	f	Secondary progressive	18	Euthanasia	2	B cell
16-019	48	m	Unknown	15	Ileus, dehydration	4	B cell
Controls							
Number	Age	Sex	Cause of death
C025	35	m	Carcinoma of the tongue				
C026	78	f	Acute myeloid leukemia				
C032	88	m	Prostate cancer with bone metastases				
C036	68	m	Cor pulmonale, heart failure, fibrosing alveolitis, coronary artery atheroma				
C037	84	m	Bladder cancer, pneumonia				
C039	82	m	Myelodysplastic syndrome, rheumatoid arthritis				
C044	67	f	Acute arrhythmia, chronic myocardial ischemia, intestinal obstruction secondary to obstructed right side femoral hernia				
C045	77	m	Cardiopulmonary degeneration, prostate cancer, old age, Alzheimer’s disease				
C048	68	m	Metastatic colon cancer				
C052	70	m	Metastatic mixed sarcoma, pulmonary emboli and myocardial infarction				
C053	66	m	Cardiac arrest				
03-117	73	f	Lung fibrosis with rhabdomyolysis and progressive renal insufficiency				
08-077	66	f	Heart failure				
10-196	60	f	Infection				
11-069	49	m	Euthanasia; Hodgkin lymphoma				

f, female; m, male; NI, no CD3/ CD20 infiltration.

### 2.2 Klüver-Barrera staining

5 μm-thick paraffin sections were deparaffinized and subsequently placed in Luxol Fast Blue (Chroma) solution at room temperature overnight. The sections were washed with distilled water and incubated in a 0.1% lithium carbonate solution for 5 min. For differentiation, the tissue was exposed to 70% isopropanol for 30 sec and washed in distilled water. After incubation in 0.2% cresyl violet solution for 2 min, the slides were rinsed in distilled water and differentiated in 96% isopropanol for 2 min. Finally, the sections were dehydrated and mounted using entellan.

### 2.3 Immunohistochemistry

After deparaffinization of human brain sections, heat-induced antigen retrieval was performed in 10 mM sodium citrate buffer (pH 6.0). The following incubation steps were done in a humified chamber and slides were washed between every incubation. Sections were blocked with either 5% milk or 5% donkey serum in tris-buffered saline with 0.5% Tween-20 (TBS-T) (blocking buffer) at room temperature for 1 h. Primary antibodies were diluted in 0.5% blocking buffer and sections were incubated in primary antibody dilution at 4°C overnight. After washing the slides with TBS-T, the appropriate secondary antibodies diluted in 0.5% blocking buffer were applied to the sections at room temperature for 2 h in the dark. For double stainings, tissue sections were washed with TBS-T and incubated with the second primary antibody at room temperature for 3 h. The corresponding secondary antibodies were diluted as mentioned earlier and incubated at room temperature for 2 h. All sections were mounted using Fluoroshield mounting medium containing 4’,6’-diamidino-2-phenylindole (DAPI) (Abcam). Details on the primary and secondary antibodies are listed in **Supplementary Table 1**. Images were acquired using a Leica DM6 fluorescence microscope equipped with LAS X software at different magnifications as indicated in the figure legends.

### 2.4 Quantification of MMP-3 staining

Tile scans were done covering either white matter (WM) lesion or corresponding normal appearing white matter (NAWM) region of every tissue section from a total number of 16 MS patients. From the 15 controls tile scans covered the WM region. Subsequently, the LAS X software algorithm was used to choose the ROIs. The number of ROIs that were analyzed was relative to the area covered by the tile scan. This resulted in 3 – 25 individual ROIs/tile scan that were quantified. Care was taken that the number of ROIs was equal between WM lesion, NAWM and the corresponding control tissue WM region. All images were acquired and analyzed in a blinded fashion. Quantification was performed using Image J (NIH). To this end, a grid with a square size of 1.5 inches was generated and placed over the images. Every square containing MMP-3^+^ cells in each ROI was counted and mean values were calculated.

### 2.5 B cell isolation and cell culture

For *in vitro* B cell studies, blood was collected from *n* = 30 healthy controls (*n* = 13 males and *n* = 17 females) between the age of 22 and 35. Inclusion criteria were an age of at least 18, written informed consent and the absence of any diagnosed diseases, in particular the absence of autoimmune and neurological diseases. In addition, participants who had shown any signs of infection two weeks before the day of the blood collection were not included in the study. The study was performed pseudonymized and approved by the ethics committee of the University of Erlangen-Nürnberg, Germany (Files 185_18B and 74_18B). Approximately 30 mL of peripheral blood per person was collected using S-Monovette^®^ tubes containing lithium heparin (Sarstedt). The blood sample was incubated with RosetteSep^™^ Human B cell enrichment cocktail (STEMCELL Technologies) and purified by Ficoll-Paque^™^ Plus (GE Healthcare) density centrifugation according to the manufacturer’s instructions. Isolated cells were counted and resuspended in complete B cell medium at a concentration of 150,000 cells/200 μL for all downstream applications. Complete B cell medium was prepared using Roswell Park Memorial Institute-1640 (RPMI-1640) medium (Sigma) containing 0.3 g/L L-glutamine, 10% fetal bovine serum (FBS) (ThermoFisher), 1% 100 IU/mL penicillin (Sigma) and 0.1 mg/mL streptomycin (Sigma) (P/S), 50 μM of β-mercaptoethanol (Sigma) and 10 mM HEPES. An aliquot of 450,000 freshly isolated B cells was separated for purity check by flow cytometry.

Purified B cells were cultured in the presence or absence of stimulation (15 ng/mL IL-2 (PeproTech) and 1 μg/mL synthetic toll-like receptor (TLR) 7/8 agonist R-848 (ENZO Life Sciences) in 96-well plates. B cells that were additionally stimulated with IL-2 and R-848 are referred to as pre-stimulated B cells and those without any stimulation as unstimulated B cells. While pre-stimulated B cells were incubated at 37°C and 5% CO_2_ for 24 h, unstimulated B cells were directly processed by adding active rMMP-3 (R&D Systems) or 4-aminophenylmercuric acetate (APMA) (Sigma). Every culture condition from every donor was plated, measured and analyzed in duplicates for all downstream applications.

### 2.6 Activation of rMMP-3

Several pro-MMPs, including pro-MMP-3, can be activated by mercurial compounds such as APMA (41). Accordingly, we used APMA to activate the pro-form of rMMP-3. rMMP-3 was activated in assay buffer (pH 7.5) at a concentration of 15 µg/mL using 1 mM APMA at 37°C for 24 h. Assay buffer was prepared using 50 mM Tris, 10 mM calcium chloride, 150 mM sodium chloride and 0.05% Brij-35. Activation of rMMP-3 was confirmed by Western blot (**Figure S1**).

### 2.7 Stimulation of B cells with active rMMP-3 or APMA

Active rMMP-3 at a titrated dose of 3 ng/well was added to pre-stimulated or un-stimulated B cells. Culturing of B cells in the presence of rMMP-3 was optimized to 20 h at 37°C and 5% CO_2_. APMA (1 µM/well) was used as a vehicle control for the different conditions.

### 2.8 Flow cytometry

#### 2.8.1 Purity check of B cells

Following every B cell isolation, the purity of the enriched population was assessed by flow cytometry. Briefly, the isolated cell suspensions were incubated with BD Horizon^™^ Fixable Viability Stain 780 (FVS780) (BD Biosciences) at 4°C for 30 min in the dark. Cells were subsequently washed, centrifuged and incubated with anti-human CD19 and CD45 antibodies at 4°C in the dark for 30 min (dilutions are listed in **Supplementary Table 1**). After washing, cells were resuspended in an appropriate volume of FACSFlow^™^ (BD Biosciences) for sample measurement. Data were acquired on a CytoFLEX S flow cytometer (Beckman Coulter) equipped with CytExpert 2.2 software. After identification of CD45^+^ leukocytes, doublets and dead cells were excluded. Subsequently, gates were set on CD19^+^ B cells. Data analysis was performed with FlowJo software (version 10.8.0) (BD Biosciences).

#### 2.8.2 B cell activation

To check the activation status of pre-stimulated or unstimulated B cells that had been treated with active human rMMP-3 or APMA for 20 h, the following cell surface markers were used: CD20, CD3, CD69, CD80 and CD86 (**Supplementary Table 1**). For live and dead cell discrimination, cells were stained with FVS780. Staining was performed as mentioned above. Details on the gating strategy used for the measurement of the B cell activation status are shown in **Figure S2**. Unstained samples, fluorescence minus one (FMO) (for CD3 staining) and isotype controls (for CD69, CD80 and CD86 staining) were also included.

#### 2.8.3 Survival of B cells

To determine cell viability of pre-stimulated and unstimulated B cells after rMMP-3 or APMA treatment, gates were set on the leukocyte population and doublets removed. After identification of CD20^+^ B cells, CD3^+^ cells were excluded and viable B cells were defined as CD20^+^CD3^-^ based on the FVS780 dye staining as mentioned above. The gating strategy is shown in **Figure S3**.

### 2.9 Antibody array and ELISA

To determine the secretion profile of cytokines by pre-stimulated B cells after treatment with rMMP-3 and APMA, a human cytokine antibody array kit with 23 targets (Abcam) was used. An equal volume of culture supernatant from rMMP-3- or APMA-treated B cells was used for the array and the assay was performed according to the manufacturer’s instructions. Based on our preliminary findings of the antibody array, quantitative analysis of IL-6 was done on B cell culture supernatant. IL-6 ELISA (ThermoFisher) was performed as per the manufacturer’s protocol.

### 2.10 RT2 profiler PCR array

Purified B cells were stimulated with R-848 and IL-2 prior to treatment and cultured as mentioned above with either active rMMP-3 or APMA. Total RNA was extracted from B cells using the RNeasy^™^ Mini kit (Qiagen) as per the manufacturer’s guidelines. In order to obtain a sufficient number of cells for the RNA extraction, B cells from 8 – 10 wells/condition were pooled together. After every extraction, the concentration and purity of the RNA yield was measured using a NanoPhotometer^®^ (Implen). Reverse transcription of RNA to cDNA was done using the High Capacity cDNA Reverse Transcriptase kit (ThermoFisher) according to the protocol suggested by the manufacturer. The expression of 44 genes associated with B cell activation and B cell receptor signaling pathways were examined in duplicates, using a customised RT2 Profiler PCR array (Qiagen). Suitable controls including housekeeping genes and controls for genomic DNA contamination were included in the array. Relative quantification of mRNA levels was performed by real-time PCR using a Roche LightCycler^®^ 480 instrument. The cycler conditions were set up following the manufacturer’s instructions. Analysis was performed using relative quantification, i.e., the gene expression profile of rMMP-3-treated B cells was determined relative to the vehicle control (APMA)-treated group. The values were normalized to the mean values of the housekeeping genes, glyceraldehyde-3-phosphate dehydrogenase (GAPDH) and transferrin receptor (TFRC).

### 2.11 Statistical analysis

Statistical analysis was perfomed using GraphPad Prism 8.0 (GraphPad Software Inc.). A Shapiro-Wilk normality test was used to test the Gaussian distribution of every data set. Differences between parametric groups were assessed using either a *t* test or a one-way ANOVA, while for non-parametric data sets, Wilcoxon test was used. The level of significance was set at 5%.

## 3 Results

### 3.1 MMP-3 expression is significantly upregulated in multiple sclerosis patients with B cell pathology

**Figure 1:**
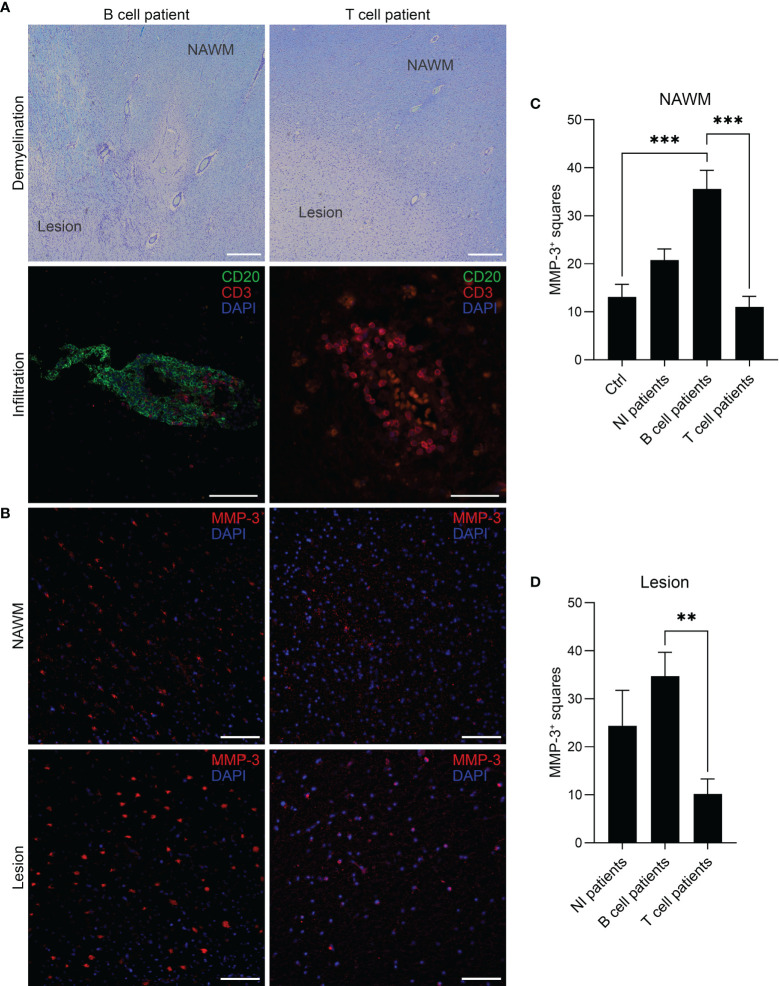
MMP-3 expression in MS brain tissue of different patient groups. **(A)** Representative images of a patient in the “B cell” and “T cell” group. Lesions and NAWM were determined by visualizing demyelinated and myelinated areas based on a Klüver-Barrera staining. Scale bars represent 500 µm. The B cell patient was characterized by the presence of both CD20^+^ B cells and CD3^+^ T cells, while the T cell patient showed infiltration of CD3^+^ T cells only. Scale bars represent 100 µm and 50 µm, respectively. **(B)** The images show MMP-3 expression in the same B cell and T cell patient (as shown in A) comparing lesion and NAWM. Scale bars represent 50 µm. **(C, D)** Quantification of MMP-3 expression in **(C)** NAWM and **(D)** lesion in different patient groups. The graph shows the number of squares containing MMP3^+^ cells by using an overlying grid. Bar graphs represent mean values + SEM. **p < 0.01, and ***p < 0.001; one-way ANOVA. NAWM, normal appearing white matter; NI, no CD3/CD20 infiltration.

**Figure 2:**
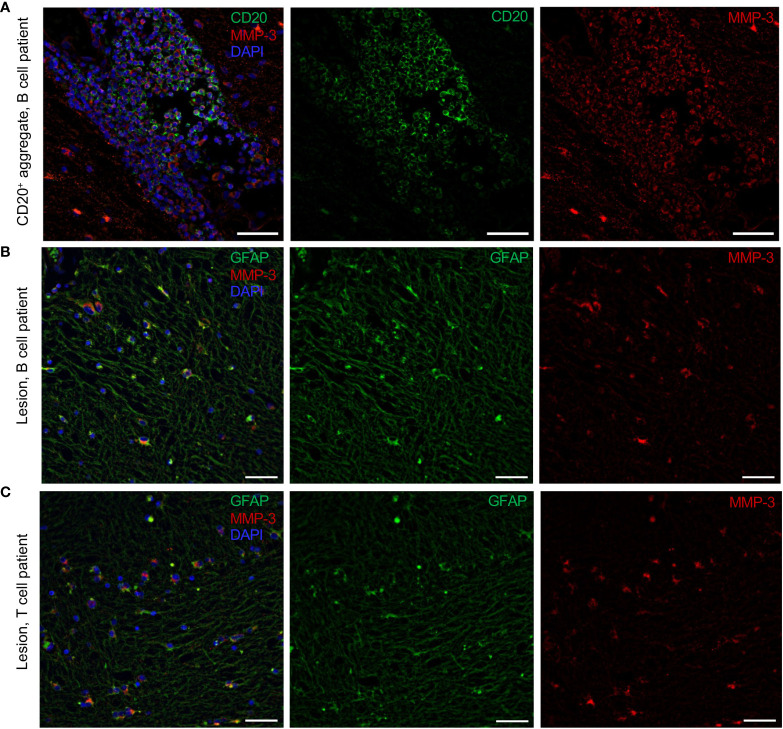
Cellular sources of MMP-3 expression in the MS brain. **(A)** Representative image of perivascular CD20^+^MMP-3^+^ B cells in MS brain sections. Parenchymal GFAP^+^MMP-3^+^ astrocytes in a **(B)** B cell patient and **(C)** T cell patient. Scale bars display 50 µm.

Immunohistochemical staining was conducted on tissue sections obtained from a cohort of 16 MS patients with a total number of 31 tissue blocks and 15 controls with one block per donor. First, MS patients were categorized into a “B cell” (*n* = 7), “T cell” (*n* = 6) or “No CD3/CD20 infiltration” (NI) (*n* = 3) group based on the presence or absence of CD20^+^ B cells and CD3^+^ T cells, respectively, confirmed by a CD20/CD3 double staining. As listed in **Table 1**, multiple blocks (1–5) were tested from patients that were categorized as “B cell positive” with at least one B cell infiltrate (counted as > 5 B cells) or B cell aggregate (counted as an aggregation of > 20 clustered B cells) in one of the blocks per patient. “T cell” patients had no B cells in the examined sections but showed pronounced CD3^+^ T cell infiltration. Representative images of a B cell aggregate in a “B cell” patient and a T cell infiltrate in a “T cell” patients are shown in **Figure 1A**. The expression pattern of MMP-3 was categorized into expression within the lesion or in the NAWM (**Figure 1B**), whereby lesions and NAWM were identified by Klüver-Barrera staining (**Figure 1A**). Although there was no difference in the expression of MMP-3 between a lesion and its corresponding NAWM, MMP-3 expression was significantly higher in the NAWM of patients in the “B cell” group compared to the “T cell” group (*p* < 0.001) and non-MS controls (*p* < 0.001), but not to the “NI” group (**Figure 1C**). Regarding the MMP-3 expression within the lesions, there was a significant difference between the “B cell” and the “T cell” group (*p* < 0.01) (**Figure 1D**). **Figure 2A** shows a representative image of the expression of MMP-3 associated with CD20^+^ B cell aggregates. GFAP^+^ astrocytes were identified as the primary source of MMP-3 in areas without CD20^+^ B cell aggregates, i.e., in lesion areas of both B cell (**Figure 2B**) and T cell patients (**Figure 2C**).

### 3.2 rMMP-3 downregulates the expression of activation and co-stimulatory markers on B cells

With the knowledge that MMP-3 is highly expressed in MS patients showing B cell pathology, the effect of MMP-3 on the activation status of B cells was determined using a panel of three pre-selected cell surface markers: CD69, which is an early surface activation marker (42), and CD80 and CD86 which are two co-stimulatory molecules that play an essential role in T cell activation (43).

Of note, while titrating the optimal APMA to MMP-3 ratio for the experiments, we noticed that an amount slightly higher than 1 µM APMA/150,000 cells/well was cytotoxic and reduced the viability of the B cells in culture. Furthermore, B cells treated with APMA alone showed alterations in their activation profile, in particular an increased expression of CD69 (**Supplementary Table 2**). Because APMA alone had an effect on the B cells, we studied the effect of rMMP on B cells relative to APMA for all the following experiments.

**Figure 3:**
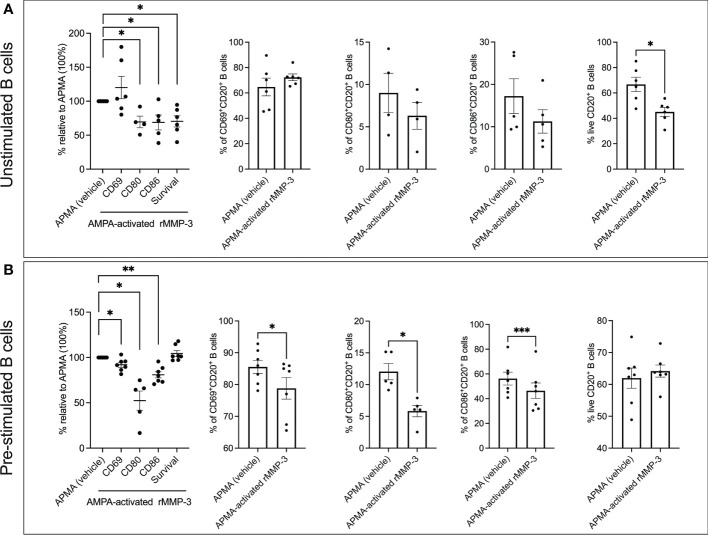
Analysis of activation markers and survival of B cells after MMP-3 stimulation. **(A)** Expression of CD69, CD80 and CD86 and viability of unstimulated (cells isolated from *n* = 4 – 6 healthy donors) B cells after treatment with APMA-activated rMMP-3 compared to APMA (vehicle) only. **(B)** Expression of CD69, CD80 and CD86 and viability of pre-stimulated (cells isolated from *n* = 5-7 healthy donors) B cells incubated with APMA-activated rMMP3 or APMA (vehicle) only. Relative expression of CD69, CD80 and CD86 and viability of either unstimulated **(A)** or pre-stimulated B cells **(B)** after treatment with rMMP-3 compared to vehicle is also shown. Graphs show mean values + SEM. *p < 0.05, **p < 0.01, ***p < 0.001; paired *t* test, Wilcoxon test. APMA, 4-aminophenylmercuric acetate.

When unstimulated peripheral blood B cells from *n* = 4 – 6 healthy donors were treated with rMMP-3 for 20 h in culture, we did not observe any significant effect on the expression of the co-stimulatory molecules (**Figure 3A**). However, B cells from a set of *n* = 5 – 7 healthy donors that were first pre-stimulated with R-848 and IL-2 and then treated with rMMP-3 demonstrated a significantly lower expression of all three surface markers (CD69 *p* = 0.018; CD80 *p* = 0.034; CD86; *p* = 0.0004) (**Figure 3B**) compared to vehicle. Interestingly, MMP-3 significantly (*p* = 0.023) reduced cell viability of unstimulated B cells, while there was no significant difference between the rMMP-3-treated and control groups in pre-stimulated B cells (**Figure 3A**). Donors, whose rMMP-3-treated B cells showed a coefficient of variation (CV) higher than 20% between the technical replicates for any condition, were excluded from the analysis.

### 3.3 B cells treated with rMMP-3 secrete significantly lower amounts of IL-6

**Figure 4:**
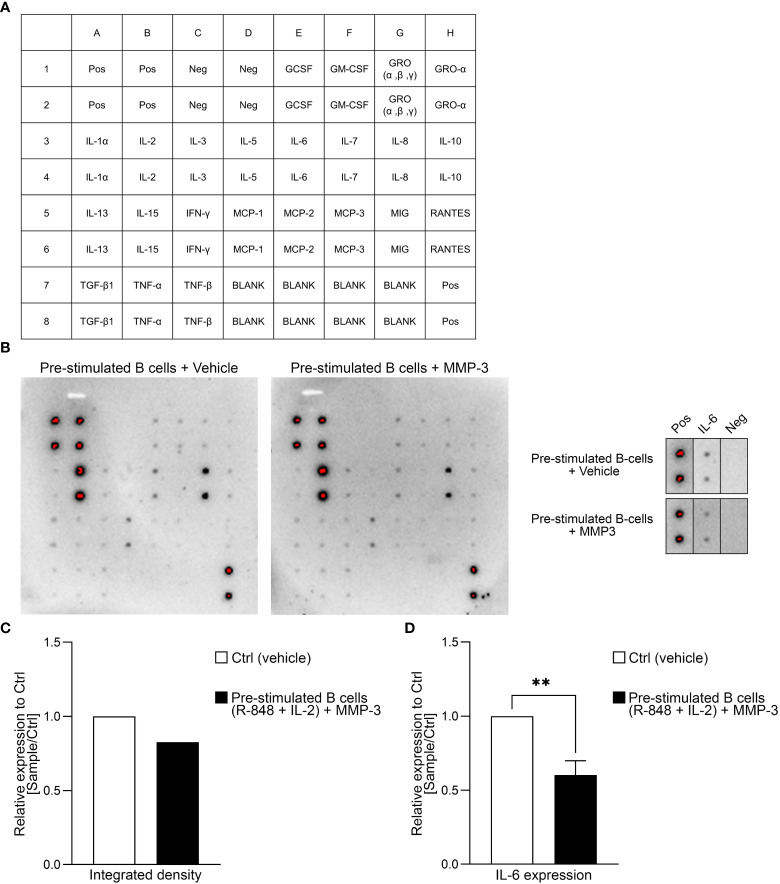
Cytokine profiling of MMP3-treated B cells. **(A)** Layout of the antibody array used for investigating cytokines. **(B)** The dot blot indicates the cytokine expression profile of pre-stimulated B cells after treatment with either MMP-3 or vehicle from one representative donor. Additionally, IL-6 production by B cells from the same donor is highlighted. **(C)** Relative amount of IL-6 production by pre-stimulated B cells treated with MMP-3 compared to vehicle was calculated by using integrated density from *n* = 1 donor, measured in duplicates. **(D)** Relative IL-6 production of pre-stimulated B cells (*n* = 9 healthy donors), measured by ELISA. Bars display mean values + SEM, **p < 0.01, paired *t* test. Pos, positive control; Neg, negative control.

To check if MMP-3 also had an effect on the cytokine profile of B cells, a dot blot antibody array was performed using the supernatants of B cells that had been pre-stimulated with R-848 and IL-2 (**Figures 4A, B**). A summary of the relative expression of the different cytokines in the rMMP-3-treated group compared to vehicle is included as **Supplementary Table 3**. The integrated density of the dot blot shows downregulation of the cytokines IL-6 and IL-8, for example, and an upregulation of tumor necrosis factor (TNF)-β from *n* = 1 donor. However, we specifically focused on the cytokine IL-6 (**Figure 4C**), which is a cytokine involved in immune responses and in chronic inflammation (44). The dot blot array was confirmed by ELISA and a significant difference between the vehicle- and rMMP-3-treated group in *n* = 9 healthy donors was observed (**Figure 4D**; *p* < 0.01).

### 3.4 rMMP-3 alters the activation, survival and proliferation profile of B cells

**Figure 5:**
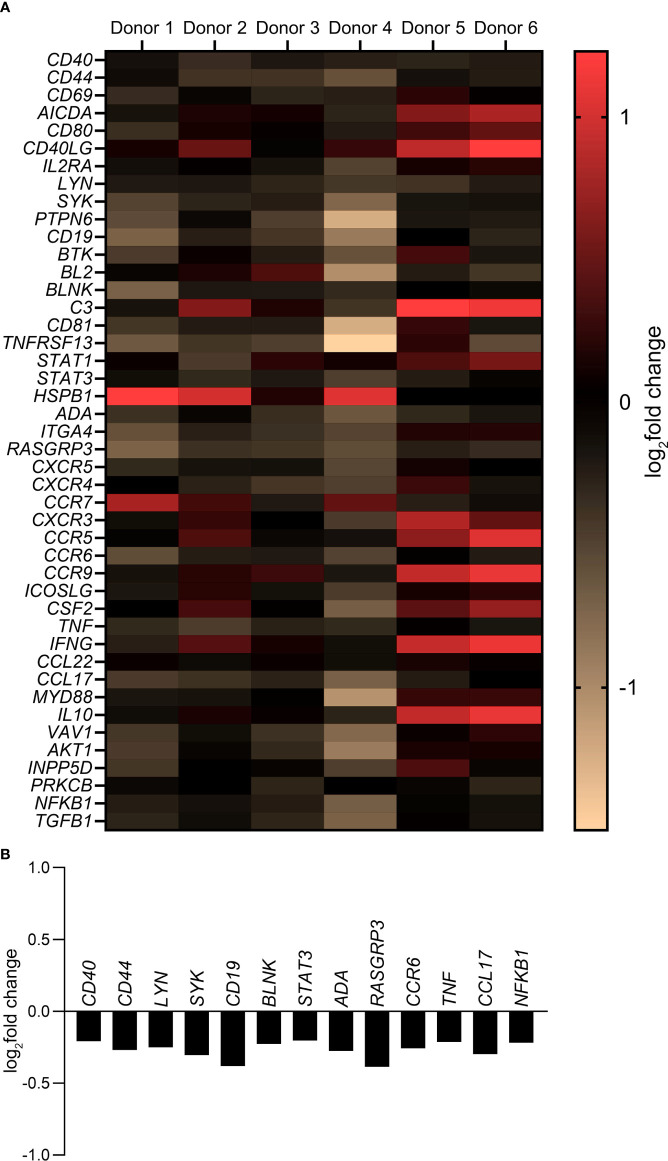
Gene expression profile of B cells after treatment with MMP-3. **(A)** Expression of B cell-related genes by pre-stimulated B cells treated with activated rMMP-3 relative to APMA in different donors (*n* = 6). B cells from 8 – 10 wells/condition/donor were pooled and measurements were done in duplicates. Values show the log_2_fold change. **(B)** The graph represents the log_2_fold change of significantly downregulated genes after MMP-3 treatment compared to the vehicle-treated group (*p* < 0.05) using paired *t* test.

A customized gene expression array with 44 genes associated with B cell activation, maturation and differentiation pathways was used to study a more global influence of MMP-3 on B cells. As shown in **Figure 5A**, a differential gene expression pattern by rMMP-3-treated B cells was apparent on the individual level (*n* = 6 healthy donors). The heat map (**Figure 5A** and **Supplementary Table 4**) summarizes the log_2_fold change comparing rMMP-3-treated B cells to the vehicle group. A total of 13 of these genes (**Figure 5B**) were significantly downregulated (*p* < 0.05) by B cells cultured in the presence of rMMP-3 compared to vehicle-treated cells, indicating that MMP-3 can modulate the B cell response at the mRNA level.

## 4 Discussion

In the present study we demonstrate that MMP-3 has the potential to modulate B cell activity not just by downregulating cell surface antigens associated with B cell activation but also by reducing expression of genes involved in B cell activation, proliferation and differentiation pathways. Furthermore, MMP-3 had an effect on IL-6 production by B cells indicating a potential role in polarizing B cells towards a more anti-inflammatory phenotype.

The first evidence of a relation between MMP-3 and B cells was demonstrated by a higher expression of MMP-3 in MS patients with CNS B cell pathology compared to those without an obvious B cell pathology. Given that lymphoid-like B cell follicles are primarily present in the progressive phase of MS (10, 14, 45), we specifically studied such a cohort of patients. Indeed, our autopsy samples provide a snapshot rather than a complete cellular overview of the brain, however, our data focuses on an increased expression of MMP-3 spatially associated with the presence of B cells. Furthermore, a few CD20^+^ B cells also co-expressed MMP-3 within B cell aggregates. Future experiments using B cell subtype-specific markers would clarify the identity of these MMP3/CD20 double positive B cells and the functional relevance of this cell population within the aggregates.

In the following sets of experiments, we have demonstrated that rMMP-3 dampens the activation status of B cells and therefore also plays a critical role in the modulation of T cell activation (46). In MS, memory B cells are considered to be the main pathogenic B cell subtype (47–49). In this context, it has been shown that memory B cells can function as potent antigen presenting cells (APCs) (50) with proinflammatory tendencies including their capacity to express high levels of proinflammatory cytokines including GM-CSF, TNF-α and lymphotoxin (51). Class-switched memory B cells not only populate the cerebrospinal fluid (CSF) of MS patients (52) but are also found within the brain parenchyma (53) with studies demonstrating that the increased accumulation of class-switched memory B cells in the CSF also correlates with the intrathecal synthesis of IgG (54–56).

Accordingly, we investigated the effects of rMMP-3 on B cells stimulated with R-848 and IL-2 as activators that trigger efficient and selective proliferation and differentiation of memory B cells (57). Additionally, R-848 is known to be a potent B cell activator (58) that can induce the synthesis of increased amounts of cytokines and antibody production in B cells (59), while IL-2 promotes the proliferation of activated B cells (60–62). We focused on the effect of MMP-3 on the activation status of B cells based on the rationale that B cells found within the CNS/CSF compartment in progressive MS patients are found to be of an activated phenotype (8, 15, 49, 63). Our data indicate that MMP-3-mediated downregulation of activation markers (i.e., of CD69, CD80 and CD86) was significant in B cells pre-stimulated with R-848 and IL-2. To understand why there is a pronounced effect of MMP-3 on pre-stimulated (memory) B cells, a more detailed flow cytometric characterization needs to be performed in future studies to allow us to confirm the nature of the effect of MMP-3 on the different subtypes of B cells in greater detail.

CD69 has been identified as a B cell marker associated with MS (64) whereas CD80 and CD86 are costimulatory molecules that are involved in immune regulation and B cells expressing CD80/CD86 have been shown to drive pathogenesis in autoimmunity (65, 66). While CD86 initiates immune reactions, CD80 is known to be the more important costimulatory molecule during chronic inflammation (67). Both CD80 and CD86 are over-expressed on B cells in patients with MS (68, 69). On the one hand, CD80 expression by B cells was significantly increased in the CSF of stable relapsing-remitting MS patients (70) and on the other hand, this increase was linked to disease exacerbations (71). Furthermore, it was suggested that an increase in CD80 on a subpopulation of activated memory B cells may be central to the development of autoimmunity (72, 73). Our data therefore indicate a therapeutic potential of the interaction between MMP-3 and B cells where MMP-3 dampens the overall activation status of B cells *via* modulation of the co-stimulatory molecules CD80/CD86.

Another effect of MMP-3 on B cells was the significant decrease in the production of IL-6, which is a proinflammatory multifunctional cytokine that promotes T-helper cell differentiation (74, 75). The therapeutic benefit of neutralizing the effects of IL-6 has already been suggested for several autoimmune diseases including systemic lupus erythematosus (SLE) and rheumatoid arthritis (RA) (76). Furthermore, a pathogenic role of IL-6 producing B cells in MS has been described (77). However, future experiments should also study the effect of MMP-3 on other B cell-secreting cytokines using a multiplex platform.

Another result of our study was the effect of MMP-3 on B cells at the mRNA level. CD40, a co-stimulatory receptor protein that is required for the generation of germinal centers and isotype switching (78), was significantly downregulated in B cells treated with MMP-3 compared to vehicle. Other genes that were significantly affected by MMP-3 included *LYN* (79) and *SYK* (80) that are known to be involved in the survival of memory B cells, in addition to *STAT3* (81)/*BLNK* (82) and *RASGRP3* (83) which play a crucial role in B cell activation and maturation. The expression level of *CD44*, which is a cell surface glycoprotein involved in a number of signal transduction events (84), was also reduced in response to MMP-3 stimulation.

It is known that through their proteolytic activity, MMPs play a crucial role in regulating signaling pathways that control cell activation and survival. However, in this case, precisely which cellular pathways are induced in B cells in a MMP-3-dependent manner or the nature of this signaling cascade remains unknown. Our findings call for the need to re-examine or discover novel substrates for MMP-3 which may include cell-associated molecules like chemokines and cell receptors expressed by B cells (85) that may explain how MMP-3 modulates B cell activity. Additionally, investigating the effect of MMP-3 on the activation status of B cells from MS patients who have not undergone any treatment with immunomodulatory drugs would strengthen the relevance of our preliminary findings.

## Data availability statement

The original contributions presented in the study are included in the article/**Supplementary Material**. Further inquiries can be directed to the corresponding author.

## Ethics statement

The studies involving human participants were reviewed and approved by the Department of Pathology, Amsterdam University Medical Center, by the Ethics Committee of the University of Würzburg and the University of Erlangen-Nuremberg, Germany. All patients/participants provided their written informed consent to participate in this study.

## Author contributions

RC and VS participated in the study design, performed all experiments, analyzed and interpreted the data and wrote the manuscript. SJ, MM and SA assisted in acquiring the human samples. SA revised the manuscript for intellectual content. SK was responsible for conceptualization, funding acquisition, study design and supervision and revised the manuscript. All authors contributed to the article and approved the submitted version.

## Funding

This research was funded by a research grant from the DFG (KU2760/4-1) to Stefanie Kuerten.

## Acknowledgments

We would like to thank Stefanie Schliwa, Benedikt Kleinsasser, Julia Lukaszczyk, Birgit Blanck and Stephanie Link for providing excellent technical assistance. We would also like to thank Benjamin Odermatt and Nina Ishorst for their help with analyzing the gene array data. Finally, we would also like to extend our appreciation to Christopher M. Overall and Lydia Sorokin for sharing their expertise on MMPs.

## Conflict of interest

The authors declare that the research was conducted in the absence of any commercial or financial relationships that could be construed as a potential conflict of interest.

## Publisher’s note

All claims expressed in this article are solely those of the authors and do not necessarily represent those of their affiliated organizations, or those of the publisher, the editors and the reviewers. Any product that may be evaluated in this article, or claim that may be made by its manufacturer, is not guaranteed or endorsed by the publisher.
