# Supplementary material for: Identification of a novel role for matrix metalloproteinase-3 in the modulation of B cell responses in multiple sclerosis
Source: Front Immunol. 2022 Oct 26;13:1025377. doi: 10.3389/fimmu.2022.1025377 (PMC9644161; doi:10.3389/fimmu.2022.1025377)
Supplement: Supplementary file 1 [file DataSheet_1.docx]

Supplementary Material

Identification of a novel role for matrix metalloproteinase-3 in the modulation of B cell responses in multiple sclerosis

**Western Blot for determination of MMP-3 activity**

To confirm the successful activation of matrix metalloproteinase-3 (MMP-3) using 4-aminophenylmercuric acetate (APMA), it was used to cleave its substrate osteopontin (OPN) (1) in an enzyme-substrate reaction. Recombinant human OPN (rOPN) was incubated at a 5:1 (substrate:enzyme) ratio in 50 µL cleavage buffer. Incubation was performed at 37°C for 24 h on a shaker. Samples were diluted in 4x NuPage™ lithium dodecyl sulfate (LDS) buffer and loaded on a 12% sodium dodecyl sulfate (SDS)-polyacrylamide gel with a pre-stained protein ladder as a marker. Immediately after running the SDS-PAGE at 100V for 2 h, protein was transferred onto a nitrocellulose membrane (0.2 μm). To prove the successful transfer of protein on the nitrocellulose membrane, a Ponceau S staining was used. The membrane was de-stained using 0.1M sodium hydroxide (NaOH) and blocked with 0.5% milk in tris-buffered saline (TBS) at room temperature for 1 h. Washing steps with TBS supplemented with 0.05% Tween-20 (TBS-T) were included between all the following steps and all incubations were done on a shaker. Subsequently, the membrane was incubated with anti-OPN antibody diluted in 0.5% milk in TBS-T (1:1000) at 4°C overnight. A biotinylated anti-rabbit antibody was used as the corresponding secondary antibody (1:5000) at room temperature for 2 h. Incubation with streptavidin-horseradish peroxidase (HRP) (1:10,000) was performed at room temperature for 1 h before the proteins were visualized using Pierce™ ECL Western Blotting Substrate. Images were acquired using an iBright™ 1500 imager.

**Active MMP-3 efficiently cleaves OPN**

Western Blot results revealed several bands of OPN indicating that APMA successfully transformed the pro-form of MMP-3 to its active form, allowing active MMP-3 to cleave its substrate OPN. Active MMP-3 generates a 40 kDa and a 32 kDa OPN fragment which is further cleaved into a 25 kDa protein band and smaller protein fragments (1). Accordingly, we detected also protein bands around the same kDa. The controls displayed weaker bands around 25 kDa, suggesting that the long incubation time of 24 h and the vehicle possibly caused some self-degradation of OPN.


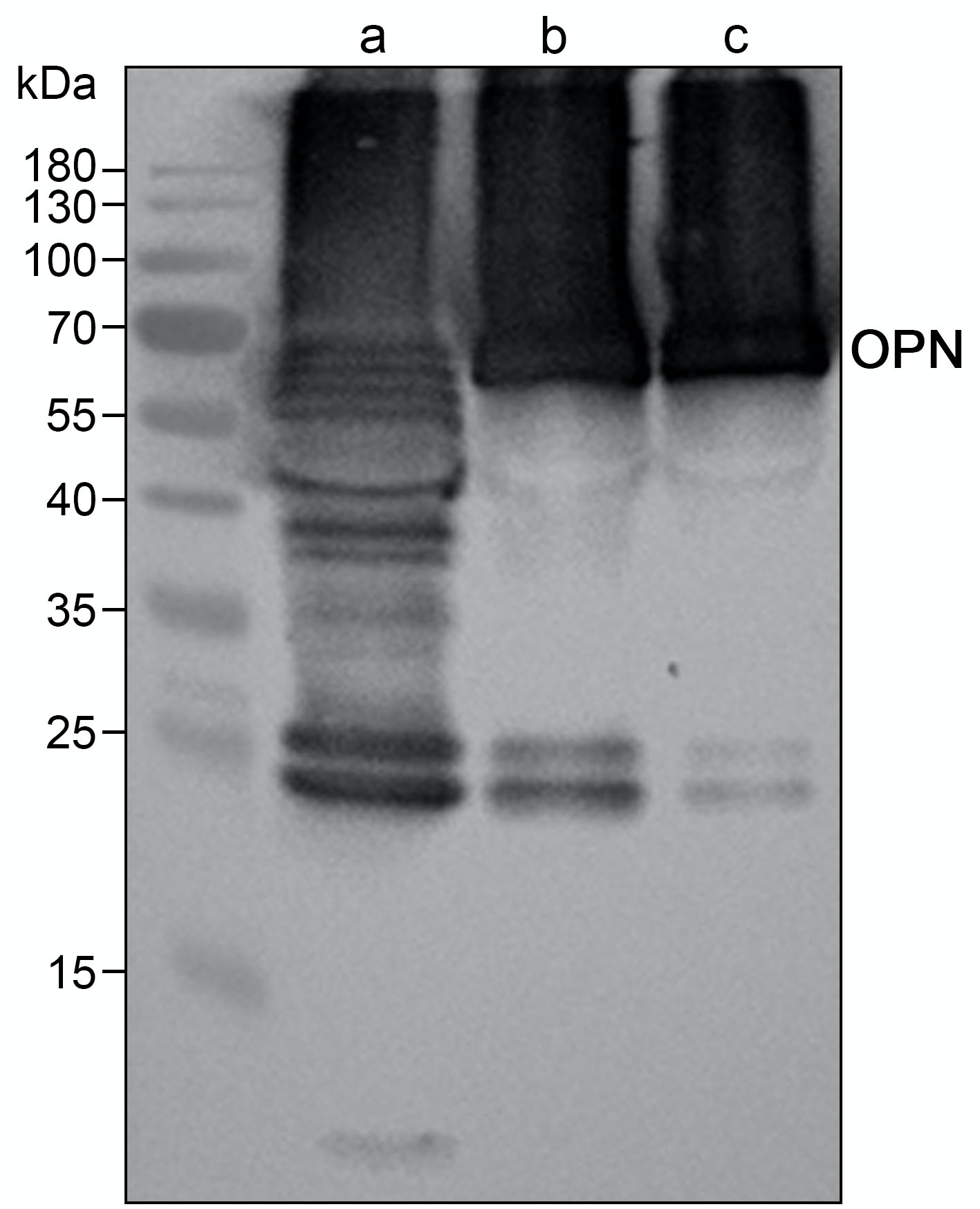


**Figure S1 Activation status of MMP-3 shown by Western Blot.**

The Western blot image displays the cleavage of rOPN by active MMP-3. Lane **a** represents the protein fragments after incubation of rOPN with active rMMP-3. In contrast, lane **b** shows rOPN incubated with vehicle (APMA) and lane **c** displays rOPN incubation in cleavage buffer only. Full length rOPN appears around 65 kDa.

**
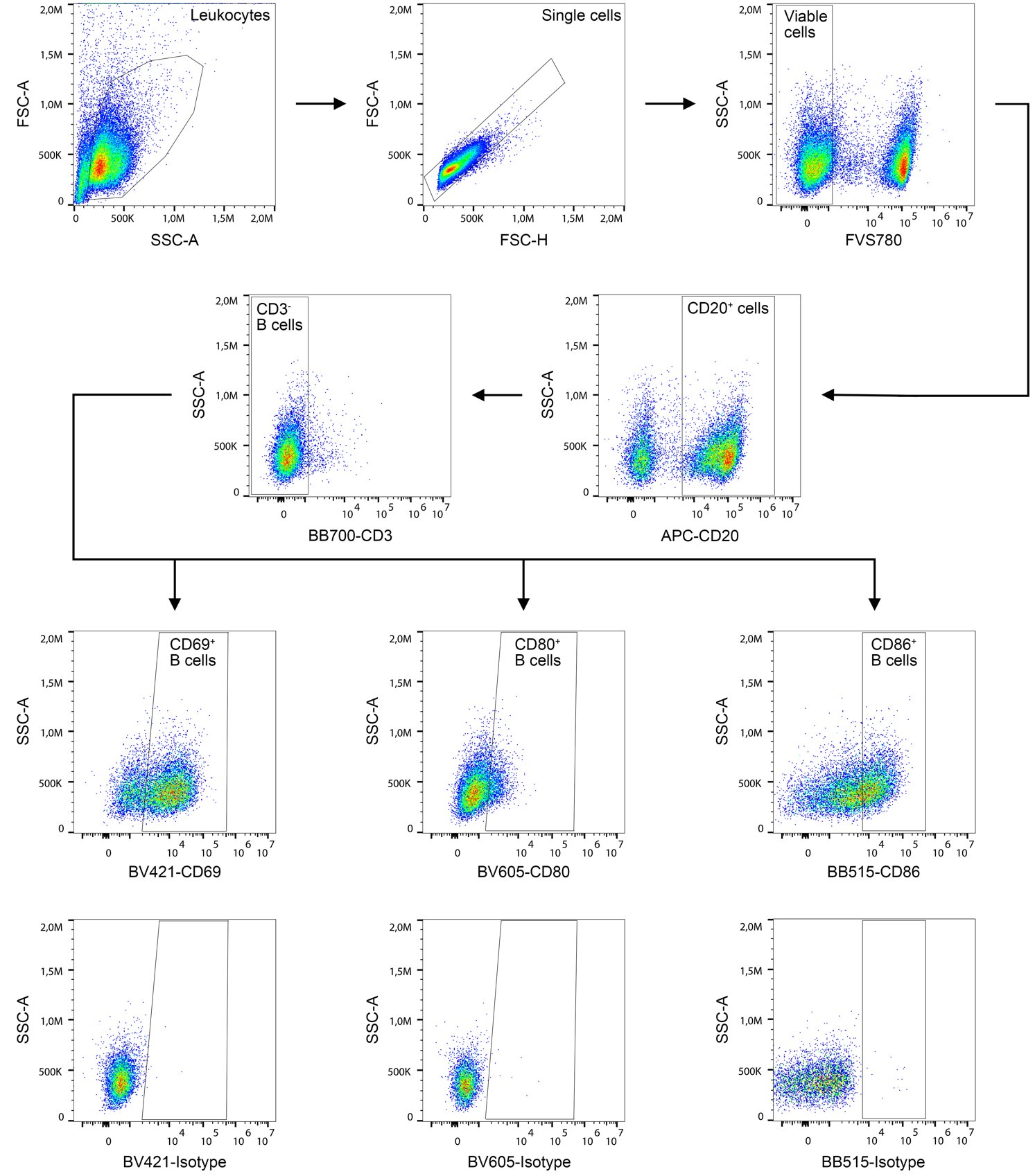
**

**Figure S2 Gating strategy for B cell activation.**

After setting the leukocyte gate, single cells were determined by forward scatter (FSC)-height (FSC-H) and FSC-area (FSC-A) and dead cells were excluded. Subsequently, CD20^+^ cells were identified and CD20^+^CD3^-^ B cells were discriminated from the CD20^+^CD3^+^ cell population, before identification of the CD69^+^, CD80^+^ and CD86^+^ B cell (CD20^+^CD3^-^) population.


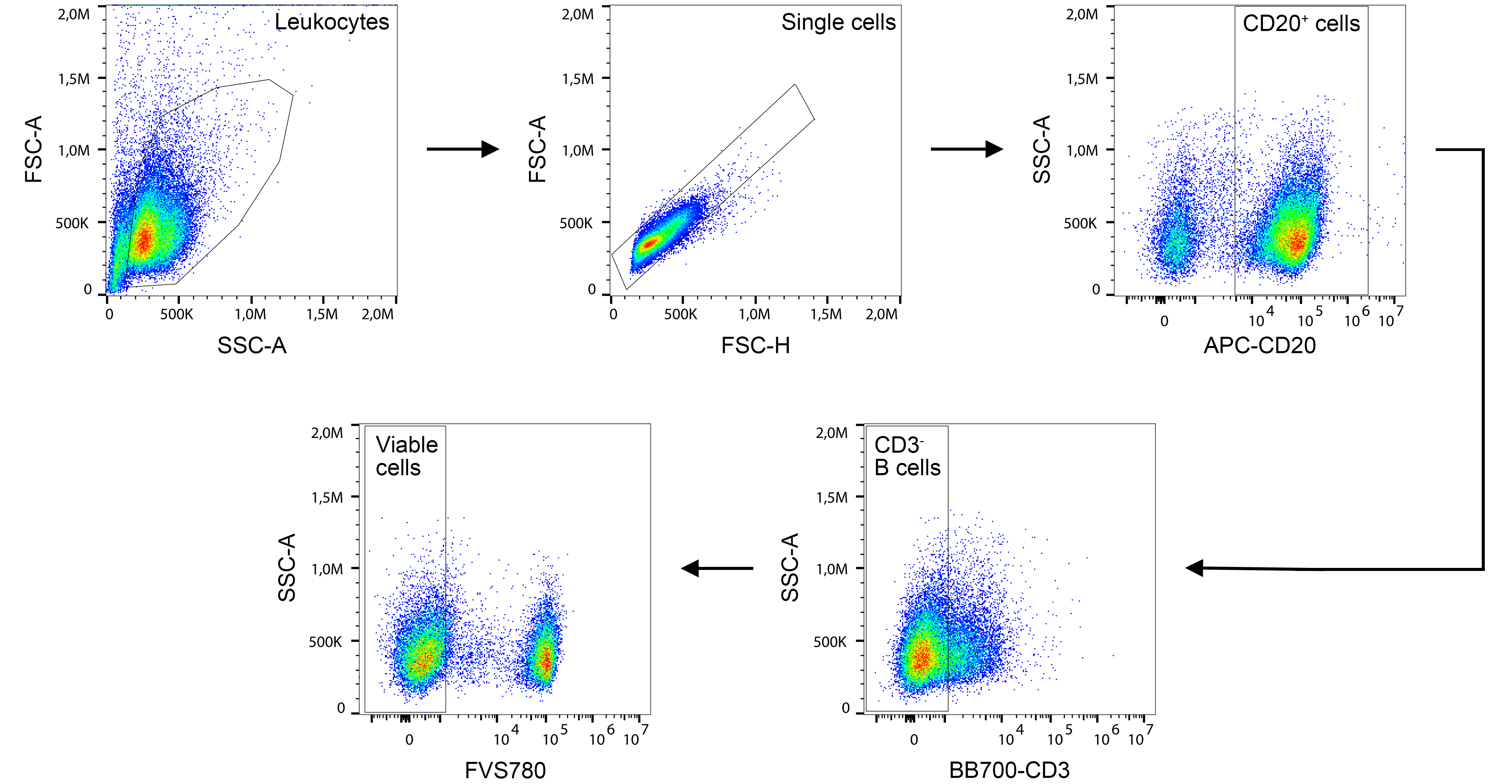


**Figure S3 Gating strategy for checking B cell viability.**

After setting the leukocyte gate, single cells were determined by forward scatter (FSC)-height (FSC-H) and FSC-area (FSC-A) and duplicates were excluded. Subsequently, CD20^+^ cells were identified and CD3^+^ cells were eliminated. The subsequent dot blot represents the viable CD20^+^CD3^-^ B cells.

**Supplementary Table 1** Antibody list and their dilutions.

| **Antibodies used for immunfluorescence** | | | |
| --- | --- | --- | --- |
| **Antibody** | **Host species** | **Clone** | **Dilution** |
| Anti-CD3 | Rabbit | SP162 | 1:150 |
| Anti-CD20 | Mouse | L26 | 1:400 |
| Anti-GFAP | Chicken | Polyclonal | 1:500 |
| Anti-MMP3 | Rabbit | Polyclonal | 1:100 |
| **Antibodies and their isotype controls used for flow cytometry** | | | |
| BB700 Anti-CD3 | Mouse | SK7 | 1:20 |
| APC Anti-CD19 | Mouse | HIB19 | 1:5 |
| APC Anti-CD20 | Mouse | 2H7 | 1:5 |
| BB515 Anti-CD45 | Mouse | HI30 | 1:20 |
| BV421 Anti-CD69 | Mouse | FN50 | 1:20 |
| BV605 Anti-CD80 | Mouse | 2D10.4 | 1:40 |
| BB515 Anti-CD86 | Mouse | FUN-1 | 1:20 |
| BV421 IgG2a | Mouse | G155-178 | 1:20 |
| BV605 IgG1 | Mouse | X40 | 1:20 |
| BB515 IgG1 | Mouse | X40 | 1:20 |

**Supplementary Table 2** Comparison between the different conditions and their effect on the activation status of B cells.

| **CD20^+^CD3^-^ B cells** | **CD69^+^ (%)** | **CD80^+^ (%)** | **CD86^+^ (%)** | **Viability (%)** |
| --- | --- | --- | --- | --- |
| Representative Donor 1 | | | | |
| Unstimulated only | 2.41 | 19.52 | 1.9 | 77.65 |
| Unstimulated + APMA | 89.52 | 1.405 | 4.93 | 55.9 |
| Unstimulated + activated rMMP3 | 71.82 | 1.41 | 1.6 | 48.8 |
| Representative Donor 1 | | | | |
| Pre-stimulated only | 29.84 | 18.16 | 44.76 | 62.9 |
| Pre-stimulated + APMA | 78.08 | 15.14 | 45.19 | 54.2 |
| Pre-stimulated + activated rMMP-3 | 67.43 | 2.53 | 31.89 | 63.95 |

**Supplementary Table 3** Integrated density of dot blot array.

| **Relative expression to Ctrl**  **[Sample/Ctrl]** | **Name of cytokine** |
| --- | --- |
| 1.046 | GCSF |
| 0.153 | GM-CSF |
| 0.075 | GRO |
| Not measurable | GRO-α |
|  | IL-1α |
| 0.903 | IL-2 |
| 1.090 | IL-3 |
| Not measurable | IL-5 |
| 0.826 | IL-6 |
| 0.634 | IL-7 |
| 0.622 | IL-8 |
| Not measurable | IL-10 |
|  | IL-13 |
|  | IL-15 |
|  | IFN-γ |
| 0.473 | MCP-1 |
| 0.913 | MCP-2 |
| 0.964 | MCP-3 |
| 1.124 | MIG |
| Not measurable | RANTES |
| 1.765 | TGF-β1 |
| 2.320 | TNF-α |
| 4.174 | TNF-β |

**Supplementary Table 4** Functions of significantly downregulated genes.

| **Genes** | **Function** |
| --- | --- |
| *CD40* | Required for the generation of germinal centers and isotype switching (2) |
| *CD44* | An activation marker which exhibits stimulatory as well as migratory capacities (3) |
| *LYN* | Plays a critical role in B cell receptor signaling (4) |
| *SYK* | Key mediator for B cell receptor signaling (5) |
| *CD19* | B cell-specific molecule involved in the survival and proliferation of B cells (6) |
| *BLNK* | A scaffold protein which is an essential component of the B cell receptor signaling pathway and is required for optimal B cell development (7) |
| *STAT3* | Required for B cell maturation (8) |
| *ADA* | Plays an essential role in controlling autoreactive B cell counter selection (9) |
| *RASGRP3* | Involved in B cell development and activation (10) |
| *CCR6* | Controls the positioning of memory B cells (11) |
| *TNF* | Important function in promoting CD4^+^ T cell expansion (12) |
| *CCL17* | Known to control leukocyte migration and maintenance of T_H_17 cells (13) |
| *NFKB1* | Required for B cell development (14) |

**Supplementary references**

1. Agnihotri R, Crawford HC, Haro H, Matrisian LM, Havrda MC, Liaw L. Osteopontin, a novel substrate for matrix metalloproteinase-3 (stromelysin-1) and matrix metalloproteinase-7 (matrilysin). *J Biol Chem*. 2001; 276(30):28261-28267. DOI: 10.1074/jbc.M103608200
2. Elgueta R, Benson MJ, de Vries VC, Wasiuk A, Guo Y, Noelle RJ. Molecular mechanism and function of CD40/CD40L engagement in the immune system. *Immunol Rev*. 2009; 229(1):152–172. doi: 10.1111/j.1600-065X.2009.00782.x
3. Flynn KM, Michaud M, Madri JA. CD44 deficiency contributes to enhanced experimental autoimmune encephalomyelitis: a role in immune cells and vascular cells of the blood-brain barrier. *Am J Pathol*. 2013; 182(4):1322-1336. doi: [10.1016/j.ajpath.2013.01.003](https://doi.org/10.1016/j.ajpath.2013.01.003)
4. Xu Y, Harder KW, Huntington ND, Hibbs ML, Tarlinton DM. Lyn tyrosine kinase: accentuating the positive and the negative. *Immunity*. 2005; 22(1):9-18. doi: [10.1016/j.immuni.2004.12.004](https://doi.org/10.1016/j.immuni.2004.12.004)
5. Ackermann JA, Nys J, Schweighoffer E, McCleary S, Smithers N, Tybulewicz VL. Syk tyrosine kinase is critical for B cell antibody responses and memory B cell survival. *J Immunol.* 2015; 194(10):4650-4656. doi: [10.4049/jimmunol.1500461](https://doi.org/10.4049/jimmunol.1500461)
6. Wang K, Wei G, Liu D. CD19: a biomarker for B cell development, lymphoma diagnosis and therapy. *Exp Hematol Oncol.* 2012; 1(1):36. doi: [10.1186/2162-3619-1-36](https://doi.org/10.1186/2162-3619-1-36)
7. Fu C, Turck CW, Kurosaki T, Chan AC. BLNK: a central linker protein in B cell activation. *Immunity*. 1998; 9(1):93-103. doi: [10.1016/s1074-7613(00)80591-9](https://doi.org/10.1016/s1074-7613(00)80591-9)
8. Oladipupo FO, Yu CR, Olumuyide E, Jittaysothorn Y, Choi JK, Egwuagu CE. STAT3 deficiency in B cells exacerbates uveitis by promoting expansion of pathogenic lymphocytes and suppressing regulatory B cells (Bregs) and Tregs. *Sci Rep*. 2020; 10(16188). doi: 10.1038/s41598-020-73093-1
9. Whitmore KV, Gaspar HB. Adenosine deaminase deficiency – More than just an immunodeficiency. *Front Immunol*. 2016; 16(7):314. doi: [10.3389/fimmu.2016.00314](https://doi.org/10.3389/fimmu.2016.00314)
10. Coughlin JJ, Stang SL, Dower NA, Stone JC. RasGRP1 and RasGRP3 regulate B cell proliferation by facilitating B cell receptor-Ras signaling. *J Immunol*. 2005; 175(11):7179-7184. doi: [10.4049/jimmunol.175.11.7179](https://doi.org/10.4049/jimmunol.175.11.7179)
11. Meitei HT, Jadhav N, Lal G. CCR6-CCL20 axis as a therapeutic target for autoimmune diseases. *Autoimmun Rev*. 2021; 20(7):102846. doi: [10.1016/j.autrev.2021.102846](https://doi.org/10.1016/j.autrev.2021.102846)
12. Popescu M, Cabrera-Martinez B, Winslow GM. TNF-α Contributes to lymphoid tissue disorganization and germinal center B cell suppression during intracellular bacterial infection. *J Immunol*. 2019; 203(9):2415-2424. doi: [10.4049/jimmunol.1900484](https://doi.org/10.4049/jimmunol.1900484)
13. Ruland C, Renken H, Kuzmanov I, Mehr AF, Schwarte K, Cerina M, et al. Chemokine CCL17 is expressed by dendritic cells in the CNS during experimental autoimmune encephalomyelitis and promotes pathogenesis of disease. *Brain Behav Immun*. 2017; 66:382-393. doi: [10.1016/j.bbi.2017.06.010](https://doi.org/10.1016/j.bbi.2017.06.010)
14. Liu T, Zhang L, Joo D, Sun SC. NF-κB signaling in inflammation. *Signal Transduct Target Ther*. 2017; 2:17023. doi: [10.1038/sigtrans.2017.23](https://doi.org/10.1038/sigtrans.2017.23)
